# Comprehensive functional characterization of murine infantile Batten disease including Parkinson-like behavior and dopaminergic markers

**DOI:** 10.1038/srep12752

**Published:** 2015-08-04

**Authors:** Joshua T. Dearborn, Steven K. Harmon, Stephen C. Fowler, Karen L. O’Malley, George T. Taylor, Mark S. Sands, David F. Wozniak

**Affiliations:** 1 Department of Internal Medicine, Washington University in St. Louis School of Medicine, St. Louis, MO, USA; 2 Department of Anatomy and Neurobiology, Washington University in St. Louis School of Medicine, St. Louis, MO, USA; 3 Department of Psychiatry, Washington University in St. Louis School of Medicine, St. Louis, MO, USA; 4 The Taylor Family Institute for Innovative Psychiatric Research, Washington University in St. Louis School of Medicine, St. Louis, MO, USA; 5 Department of Pharmacology and Toxicology, University of Kansas, Lawrence, KS, USA; 6 Department of Psychology, University of Missouri-St. Louis, St. Louis, MO, USA

## Abstract

Infantile neuronal ceroid lipofuscinosis (INCL, Infantile Batten disease) is a neurodegenerative lysosomal storage disease caused by a deficiency in palmitoyl protein thioesterase-1 (PPT1). The PPT1-deficient mouse (*Cln1*^*−/−*^) is a useful phenocopy of human INCL. *Cln1*^*−/−*^ mice display retinal dysfunction, seizures, motor deficits, and die at ~8 months of age. However, little is known about the cognitive and behavioral functions of *Cln1*^*−/−*^ mice during disease progression. In the present study, younger (~1–2 months of age) *Cln1*^*−/−*^ mice showed minor deficits in motor/sensorimotor functions while older (~5–6 months of age) *Cln1*^*−/−*^ mice exhibited more severe impairments, including decreased locomotor activity, inferior cued water maze performance, decreased running wheel ability, and altered auditory cue conditioning. Unexpectedly, certain cognitive functions such as some learning and memory capabilities seemed intact in older *Cln1*^*−/−*^ mice. Younger and older *Cln1*^*−/−*^ mice presented with walking initiation defects, gait abnormalities, and slowed movements, which are analogous to some symptoms reported in INCL and parkinsonism. However, there was no evidence of alterations in dopaminergic markers in *Cln1*^*−/−*^ mice. Results from this study demonstrate quantifiable changes in behavioral functions during progression of murine INCL and suggest that Parkinson-like motor/sensorimotor deficits in *Cln1*^*−/−*^ mice are not mediated by dopamine deficiency.

Lysosomal storage diseases (LSDs) are a group of >50 distinct disorders characterized by abnormal intracellular accumulation of undegraded substrates. The accumulation of undegraded substrates results from defective lysosomal function, often due to reduced or absent activity of a specific enzyme. The neuronal ceroid lipofuscinoses (NCLs) are a group of at least 14 distinct disorders that comprise a subset of LSDs typified by intracellular accumulation of autofluorescent storage material throughout the brain and body. The most rapidly progressing form of NCL is infantile neuronal ceroid lipofuscionosis (INCL), commonly referred to as Infantile Batten disease^1^,^2^,^3^,^4^,^5^,^6^.

Infantile NCL is associated with an autosomal recessive mutation in the *Cln1* gene which encodes palmitoyl-protein thioesterase-1 (PPT1), a lysosomal enzyme which catalyzes the cleavage of thioester linkage that attaches long-chain fatty acids (predominantly palmitate) with specific cysteine residues in polypeptides^7^,^8^. Patients with INCL appear unaffected at birth and show normal central nervous system (CNS) development until the age of 6–12 months. By 1 to 1.5 years, they exhibit a progression of symptoms including visual loss and motor impairments. Intractable seizures appear between 16 and 24 months, and death occurs as early as 6 years, although some live into their teenage years^4^,^9^,^10^.

In recent years an animal model that closely mimics human INCL has been developed by creating a PPT1-knockout mouse (*Cln1*^*−/−*^)^10^. These mice are completely deficient in PPT1 activity and exhibit retinal dysfunction, progressive motor/sensorimotor abnormalities, spontaneous seizures, and a shortened life span of approximately 8 months^10^,^11^. Additionally, this animal model recapitulates the microglial activation, loss of gamma-aminobutyric acid (GABA) neurons, apoptosis, astrocytosis, and cortical atrophy seen in the human disease^2^,^12^,^13^.

Sufficient research has accrued to confirm this knockout mouse as a useful animal model for human INCL. However, much remains unknown regarding the *Cln1*^*−/−*^ mouse, not the least of which is a more complete behavioral profile beyond testing simple motor abilities. Therefore, we performed a comprehensive behavioral characterization of the *Cln1*^*−/−*^ mouse at two ages: one cohort was tested beginning at 1 month of age, and the other was tested beginning at 5 months of age. Results of this study identified a number of behavioral deficits that will serve as important end points when studying INCL disease progression and treatment efficacy. This study also identified Parkinson-like behavioral deficits in the *Cln1*^*−/−*^ mice. This is consistent with reports showing Parkinson-like motor deficits in the NCLs^14^,^15^,^16^,^17^,^18^. However, in the current study there is no biochemical or histological evidence of alterations in dopaminergic markers in the brains of *Cln1*^*−/−*^ mice. These findings highlight the importance of biochemical and histological confirmation when making a link between LSDs and parkinsonism.

## Results

### Younger Mouse Behavior

**Figure 1:**
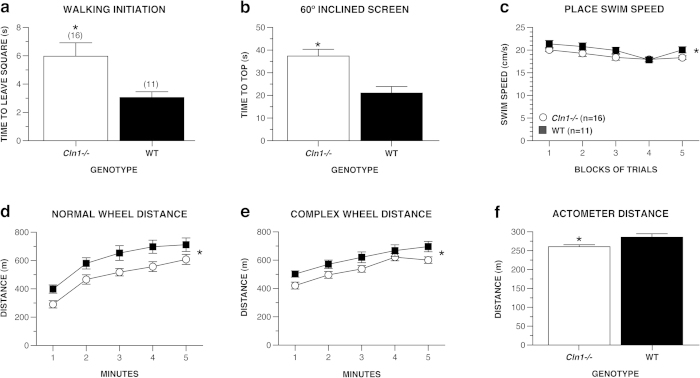
Younger *Cln1*^*−/−*^ mice exhibited mild behavioral deficits compared to WT control mice. Subtle but significant deficits were found in younger *Cln1*^*−/−*^ mice, including delayed initiation of walking (**a**), *p = 0.023, slowed climbing on a 60° inclined screen (**b**), *p = 0.001, and slower swim speeds on average across place MWM trials (**c**), *p = 0.018. *Cln1*^*−/−*^ mice also traveled a shorter distance during normal running wheel trials (**d**), *p = 0.014, complex running wheel training (**e**), *p = 0.048, and during testing in the actometer (**f**), *p = 0.017. Data are expressed as mean ± standard error, open circles represent *Cln1*^*−/−*^ mice (*n* = 16) and filled squares represent WT mice (*n* = 11).

The mice were tested on a battery of tests to assess potential deficits in cognitive and other behavioral functions in the *Cln1*^*−/−*^ mice under controlled conditions across ages representing different stages of disease progression. Results from testing the younger cohort of mice showed that there were no significant differences between *Cln1*^*−/−*^ mice and age-matched WT mice on most of the behavioral tests. However, there were subtle but statistically significant differences between the groups on a few tests measuring motor/sensorimotor performance. Specifically, *Cln1*^*−/−*^ mice showed a significant impairment in initiating movement out of a small circumscribed area (walking initiation; Fig. 1a) and were slower to climb to the top of the 60° inclined screen (Fig. 1b), both tests being part of the battery of sensorimotor measures, [*F*(1, 25) = 5.856, *p* = 0.023; *F*(1, 25) = 14.16, *p* = 0.001, respectively]. Younger *Cln1*^*−/−*^ mice also exhibited decreased swimming speed (Fig. 1c) during place trials [*F*(1, 25) = 6.36, *p* = 0.018] in the Morris water maze (MWM), but importantly there were no significant performance differences between them and the WT group with regard to path length on either type of cued trials, suggesting that the younger *Cln1*^*−/−*^ mice did not have deficits in visually-guided behavior. *Cln1*^*−/−*^ mice also traveled a shorter distance on the normal running wheel [*F*(1, 25) = 7.00, *p* = 0.014], during complex wheel training [*F*(1, 25) = 4.324, *p* = .048], and in the actometer [*F*(1, 26) = 6.53, *p* = 0.017] (Fig. 1d–f).

Notably, the younger *Cln1*^*−/−*^ mice performed similarly to WT mice on 5 of 7 sensorimotor tests and with regard to some of the cognitive measures in the MWM such as path length during place (spatial learning) trials. WT and *Cln1*^*−/−*^ mice also did not differ in terms of platform crossings, time in the target quadrant, and spatial bias during the probe trial in the MWM suggesting intact retention (data not shown). Group performances also were not different on other cognitive measures such as the contextual fear and auditory cue components during conditioned fear testing (data not shown). As such, younger *Cln1*^*−/−*^ mice were mostly indistinguishable from WT mice except for some deficits in speed of movement and distance traveled.

### Older Mouse Behavior

#### 1-h locomotor activity

**Figure 2:**
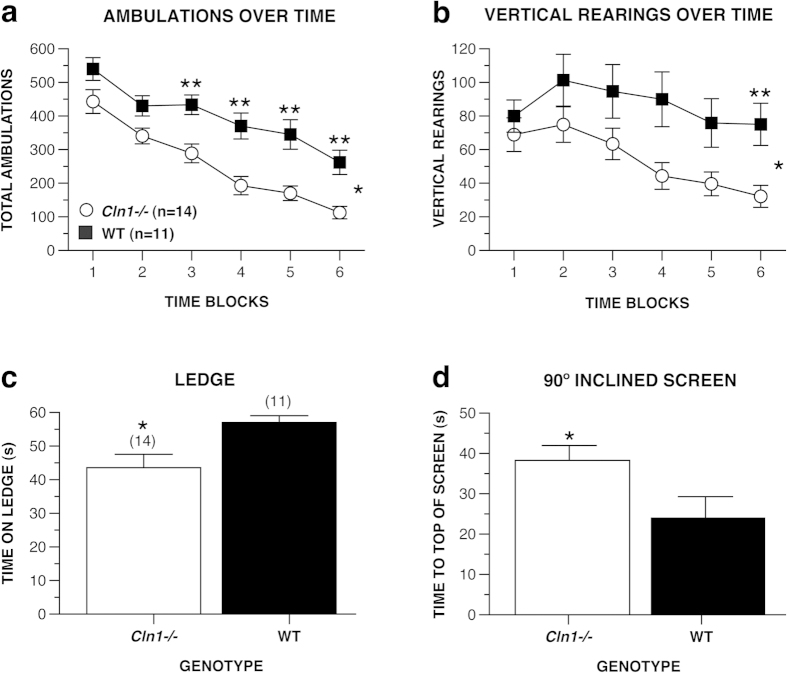
Older *Cln1*^*−/−*^ mice showed significantly reduced levels of general ambulatory activity and vertical rearing as well as motor/sensorimotor impairments relative to WT mice. *Cln1*^*−/−*^ mice demonstrated fewer total ambulations (whole body movements) (**a**), *p* = 0.001, and fewer rears (**b**), *p* = 0.034 on average across time blocks during the 1-h locomotor activity test. In addition, *Cln1*^*−/−*^ mice showed deficits on the ledge test for balance (**c**), *p* = 0.011, and 90° inclined screen test (**d**), *p* = 0.032. Data are expressed as mean ± standard error, open circles represent *Cln1*^*−/−*^ mice (*n* = 14) and filled squares represent WT mice (*n* = 11), *p < 0.05 between groups, **p < 0.005 between groups at a single time block.

To assess general activity levels and exploratory behaviors in response to novel environmental stimuli, as well as other processes such as habituation and emotionality (center variables), the mice were evaluated on a 1-hr locomotor activity test. In contrast to the younger cohort results, older *Cln1*^*−/−*^ mice differed significantly from WT mice on every performance variable of this test. For example, analyses of the total ambulations (whole body movements) and vertical rearing data (Fig. 2a,b) produced significant main effects of genotype for each variable, [*F*(1, 23) = 15.11, *p* = 0.001 and *F*(1, 23) = 5.10, *p* = 0.034 respectively], indicating that the *Cln1*^*−/−*^ mice exhibited significantly decreased levels for both variables over time. In addition, *Cln1*^*−/−*^ mice traveled shorter distances in the peripheral zone [*F*(1, 24) = 6.95 *p* = 0.015]. With regard to the emotionality variables, the *Cln1*^*−/−*^ mice traveled decreased distances in the center zone [*F*(1, 24) = 22.62 *p* < 0.0005], entered the center zone less frequently [*F*(1, 24) = 26.63 *p* < 0.0005], and spent less time in the center zone [*F*(1, 24) = 4.49 *p* = 0.045) compared to WT mice (data not shown). Lastly, older *Cln1*^*−/−*^ mice spent significantly more time resting during the 1 hr test than the WT mice, [*F*(1, 24) = 19.77, *p* < 0.0005] (not shown).

#### Sensorimotor Battery

Patients with INCL present with significant pathology in motor cortex, somatosensory cortex, and cerebellum; motor deficits are a well-established symptom of this disease. Our mice were evaluated on various sensorimotor tests to measure analogous deficits at different ages. One-way ANOVAs yielded significant genotype effects for the ledge, [*F*(1, 24) = 7.70, *p* = 0.011], and the 90° inclined screen, [*F*(1, 24) = 5.20, *p* = 0.032], tests showing that *Cln1*^*−/−*^ mice spent less time balancing on the ledge and required more time to climb to the top of the 90° screen compared to WT mice (Fig. 2c,d). There were no differences between groups on any of the other sensorimotor measures.

#### Morris Water Maze

**Figure 3:**
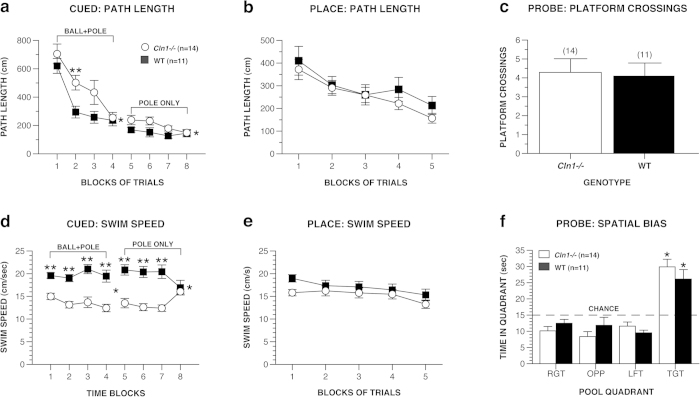
Older *Cln1*^*−/−*^ mice demonstrated intact spatial learning ability during place MWM trials although they showed deficits during the preceding cued trials. During cued trials, *Cln1*^*−/−*^ mice took significantly longer paths to find the platform on average across blocks of trials whether it was cued by the pole + ball or the pole alone (**a**), *p* < 0.0005 for both, and they swam significantly more slowly compared to WT controls regardless of how the platform was cued (**d**), *p* < 0.0005. However, during place trials, these deficits were no longer apparent. Older *Cln1*^*−/−*^ mice required comparable path lengths in navigating to the platform (**b**), and swam at similar speeds compared to WT mice (**e**). During the MWM probe trial, groups performed similarly with regard to number of platform crossings (**c**) and spatial bias for the target quadrant that had contained the platform (**f**). Data are expressed as mean ± standard error, open circles represent *Cln1*^*−/−*^ mice (*n* = 14) and filled squares represent WT mice (*n* = 11), *p < 0.05 between groups and **p < 0.005 between groups at a single time block for the cued path length and swimming speeds, *p < 0.006 for comparisons between the target quadrant and each of the other pool quadrants.

Little is known about the cognitive capabilities of INCL patients, and they are difficult to study under controlled conditions. In an effort to provide information on the intactness of cognitive abilities as a function of disease progression in our INCL mouse model, we assessed spatial learning and memory in the mice in the MWM at different ages. During traditional cued trials (ball + pole), significant genotype effects indicated that the *Cln1*^*−/−*^ mice exhibited longer path lengths [F(1, 23) = 7.56, *p* = 0.011], and slower swimming speeds [F(1, 23) = 33.00, *p* < 0.0005), to find the escape platform compared to WT mice (Fig. 3a,d). The *Cln1*^*−/−*^ mice also had greater escape latencies in navigating to the platform [F(1, 23) = 36.62, *p* < 0.0005] (data not shown). The large differences in swimming speeds make escape path length a more appropriate variable for evaluating performance instead of latency. Contrasts conducted between block 1 versus block 4 in each group indicated that both *Cln1*^*−/−*^ and WT mice showed significant (*p* < 0.0005) improvement in escape path length across blocks of trials, providing evidence of cued learning. For the cued trials when the platform location was marked only by a pole to decrease the salience of the cue and provide a more difficult test for visually-guided behavior (second 4 trial blocks), a significant genotype effect was found for path length, [*F*(1, 23) = 8.97, *p* = 0.006], indicating that the *Cln1*^*−/−*^ mice required greater distances in navigating to the platform. A significant genotype effect, [*F*(1, 23) = 23.88, *p* < 0.0005], and a significant genotype x trial block interaction [*F*(3, 69) = 11.53, *p* < 0.0005] was found for swimming speeds, with subsequent pair-wise comparisons showing that *Cln1*^*−/−*^ mice swam more slowly compared to WT mice during trial blocks 5, 6, and 7 (p < 0.0005), but performed similarly to WT mice during trial block 8.

During place (spatial learning) trials, there were no significant main or interaction effects involving genotype for escape path length (Fig. 3b) or latency (not shown). Comparing performance during block 1 versus block 5 in each group indicated that both *Cln1*^*−/−*^ and WT mice required shorter path lengths (*p* < 0.0005) and decreased latency to find the platform (*p* < 0.0005) (not shown) across blocks of trials, providing evidence for spatial learning acquisition in both groups. In contrast to the cued data, no significant main or interaction effects involving genotype were found with regard to the swimming speed data (Fig. 3e). During the probe (retention) trial, both groups exhibited a spatial bias for the target quadrant thus providing further documentation that both groups learned the location of the hidden platform (Fig. 3c). Specifically, each group spent significantly more time in the target quadrant that had contained the platform compared to the times spent in each of the other quadrants (*p* < 0.0005). In addition, no significant differences were observed between the groups with regard to platform crossings (Fig. 3f) or time spent in the target quadrant.

#### Visual acuity

**Figure 4:**
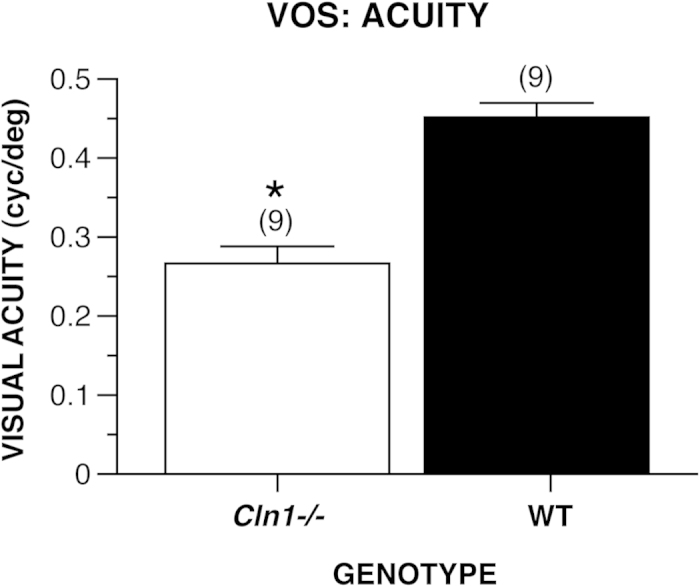
7-month-old *Cln1*^*−/−*^ mice exhibited a significantly impaired visual acuity compared to WT mice. The threshold of grating (cycles per degree) of light/dark rotating lines at which older *Cln1*^*−/−*^ mice (*n* = 9) failed to display an optokinetic response was significantly lower than that of WT mice (*n* = 9), *p < 0.0005. Data are expressed as mean + standard error.

Since loss of vision occurs in patients with INCL, and since the older *Cln1*^*−/−*^ mice exhibited impaired performance on the two types of MWM cued trials, we evaluated an independent, 7-month old cohort of *Cln1*^*−/−*^ and WT control mice on the virtual optomotor system (VOS) to determine visual (grating) acuity thresholds. The results indicated that the *Cln1*^*−/−*^ group displayed a significantly lower acuity (grating) threshold for the optokinetic response compared to WT mice, t(16) = 6.51, *p* < 0.0005, signifying impaired visual system function at 7 months (Fig. 4).

#### Normal and complex running wheel

**Figure 5:**
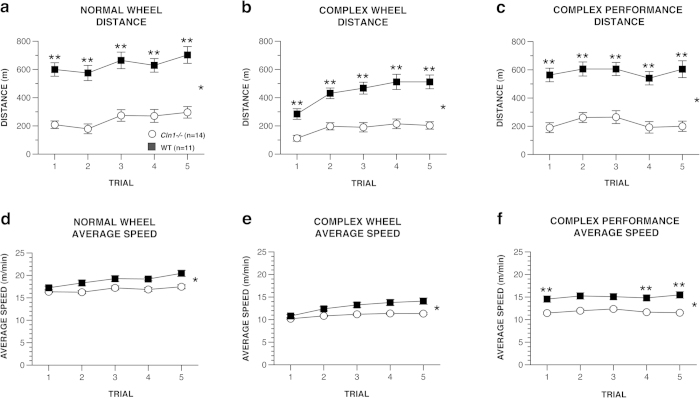
Older *Cln1*^*−/−*^ mice exhibited running wheel performance deficits for all test variables. Specifically, *Cln1*^*−/−*^ mice traveled a significantly shorter distance during normal (baseline) wheel running (**a**), complex wheel training (**b**), and complex wheel performance (**c**) compared to WT mice, (genotype effects: *p < 0.0005 for all variables). Also, *Cln1*^*−/−*^ mice ran at significantly slower average speeds (genotype effects: *p < 0.05) during the same test conditions (**d–f**). Data are expressed as mean ± standard error, open circles represent *Cln1*^*−/−*^ mice (*n* = 14) and filled squares represent WT mice (*n* = 11), **p < 0.005 between-groups (pair-wise) comparisons for individual daily trials.

Performance on the normal and complex running wheels was evaluated to measure abilities of *Cln1*^*−/−*^ mice in a motor learning paradigm. The complex wheel involves removing rungs of the wheel at different intervals creating unequal spaces between rungs, thus creating a sensitive test that requires fine motor control and integration between the forelimbs and hindlimbs. The mice received daily 1 hr trials during both the normal and complex wheel-running conditions. During baseline testing on the normal running wheel, *Cln1*^*−/−*^ mice traveled a shorter distance [*F*(1, 23) = 44.44, *p* < 0.0005] and at a slower average speed [*F*(1, 16) = 5.73, *p* = 0.029] than WT mice (Fig. 5a,d). In addition, WT mice reached a higher maximum speed [*F*(1, 23) = 17.27, *p* < 0.0005) compared to *Cln1*^*−/−*^ mice (not shown).

Similar significant effects of genotype were found for distance traveled, [*F*(1, 23) = 30.89, *p* < 0.0005], average speed, [*F*(1, 14) = 5.143, *p* = 0.040], and maximum speed, [*F*(1, 23) = 15.070, *p* = 0.001], during training on the complex wheel (first 5 days). This documents that the *Cln1*^*−/−*^ mice traveled a shorter distance (Fig. 5b), had a slower average running speed (Fig. 5e), and achieved a slower maximum speed (not shown) compared to WT mice on average across trials. Both *Cln1*^*−/−*^ and WT mice showed a significant increase in distance traveled during trial 1 versus trial 5, (Fig. 5b; *p* < 0.001), indicating motor learning occurred in both groups. Although there was a significant genotype effect for average speed during training on the complex wheel (Fig. 5e), the groups did not differ significantly on any given trial, and only WT mice exhibited an increase in average speed from trial 1 to trial 5, (*p* < 0.0005).

The pattern of between-group differences was essentially repeated during performance testing on the complex wheel (second 5 days). Overall, *Cln1*^*−/−*^ mice ran a shorter total distance in the wheel, [*F*(1, 23) = 36.92, *p* < 0.0005] (Fig. 5c), at a slower average speed, [*F*(1, 17) = 11.99, *p* = 0.003] (Fig. 5f), and reached a slower maximum speed, [*F*(1, 23) = 25.11, *p* < 0.0005] (not shown) compared to WT mice. Neither group showed a reliable increase in average speed or maximum speed from trial 1 to trial 5 (data not shown).

#### Conditioned fear

**Figure 6:**
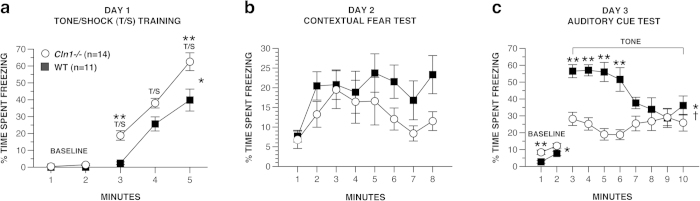
Contextual fear conditioning was not impaired in older *Cln1*^*−/−*^ mice although they showed significant impairment in auditory cue conditioning and an altered freezing response to tone-shock (T/S) training compared to WT mice. *Cln1*^*−/−*^ mice froze significantly more than WT mice during baseline, *p = 0.028, and T/S training on day 1 of conditioned fear testing (**a**), *p < 0.0005. Groups performed similarly during the contextual fear test on day 2 (**b**). *Cln1*^*−/−*^ mice froze significantly more than WT mice during the altered context baseline on day 3, *p = 0.002, but then exhibited significantly decreased freezing levels during subsequent minutes (**c**). Data are expressed as mean ± standard error, open circles represent *Cln1*^*−/−*^ mice (*n* = 14) and filled squares represent WT mice (*n* = 11), *p < 0.05 for genotype effects, †p < 0.0005 for genotype x minute interaction, **p < 0.005 between-groups comparisons for individual minutes.

To provide additional information on nonspatial learning and memory capabilities of our INCL model mice, we assessed their performance on a Pavlovian-based conditioned fear test which involves quantifying freezing as a behavioral response. Analysis of the data during the first 2 min of testing on day 1 to establish baseline freezing levels (Fig. 6a, baseline data) revealed that the *Cln1*^*−/−*^ mice froze significantly more than WT mice, [*F*(1, 23) = 5.49, *p* = 0.028]. Similarly, a significant genotype effect was found during the subsequent 3 min of tone-shock (T/S) training [conditioned stimulus-unconditioned stimulus (CS-US) pairings] on day 1, [*F*(1, 23) = 19.92, *p* < 0.0005] documenting increased levels of freezing in the *Cln1*^*−/−*^ mice on average across minutes (Fig. 6a, T/S data). Pair-wise comparisons showed that the *Cln1*^*−/−*^ mice had significantly elevated freezing levels during minutes 3 (*p* < 0.0005) and 5 (*p* = 0.012) compared to the WT group.

Analyses of the freezing data from the contextual fear test conducted on day 2 did not reveal any main or interaction effects involving genotype suggesting that contextual fear conditioning was relatively intact in *Cln1*^*−/−*^ mice (Fig. 6b). In contrast to these data were the differences in freezing levels observed between groups on day 3 (Fig. 6c). Specifically, *Cln1*^*−/−*^ mice exhibited significantly increased levels of freezing during the 2-min altered context baseline compared to the WT group overall, [*F*(1 ,23) = 11.96, *p* = 0.002] and also during min 1 (*p* = 0.004). Interestingly, analysis of the auditory cue data (min 3–10) on day 3 revealed very different patterns of freezing levels between the groups across the 8-min period. Specifically, a significant genotype effect, [*F*(1, 23) = 28.07, *p* < 0.0005] and genotype x minute interaction, [*F*(5, 60) = 128.78, *p* < 0.0005] indicated that differences between groups varied across the 8-min interval. Subsequent pair-wise comparisons showed that the older *Cln1*^*−/−*^ mice had significantly decreased freezing levels relative to WT controls for minutes 3, 4, 5, and 6 (*p* < 0.0005) while freezing levels were similar throughout the rest of the interval. In addition, freezing levels of the *Cln1*^*−/−*^ mice remained relatively stable across minutes, whereas the WT mice showed a dramatic decrease in freezing over time suggesting that habituation had taken place. This is documented by WT mice freezing significantly less during minute 10 compared to minute 3 (*p* < 0.0005).

#### Actometer

**Figure 7:**
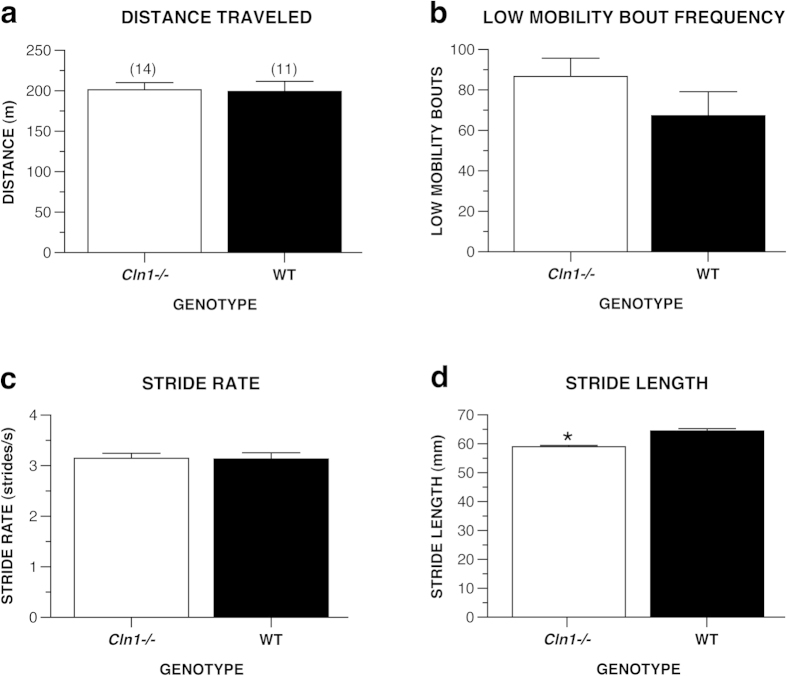
Older *Cln1*^*−/−*^ mice demonstrate a gait abnormality typically associated with murine models of parkinsonism. During testing in the force-plate actometer apparatus, *Cln1*^*−/−*^ mice (n = 14) traveled a similar distance (**a**), exhibited a similar number of low mobility bouts (**b**), and moved with similar stride rates (**c**) relative to WT mice (n = 11). In contrast, *Cln1*^*−/−*^ mice ambulated across the test field using shorter stride lengths compared to WT mice (**d**), *p < 0.0005. Data are expressed as mean ± standard error.

A growing body of literature connects Parkinson-like symptoms to LSDs. To assess whether spontaneously-occurring gait abnormalities and/or Parkinson-like behavior were present in the INCL model mice, we analyzed several movement-related variables by testing the mice in the force-plate actometer. There were no main effects of genotype with regard to distance traveled, frequency of low mobility bouts or stride rate during the actometer test (Fig. 7a–c). However, *Cln1*^*−/−*^ mice ambulated across the test chamber using a shorter stride length compared to WT mice, [*F*(1, 23) = 37.64, *p* < 0.0005] (Fig. 7d). Shortened stride length, as measured by the actometer apparatus, is a characteristic reliably found in the MPTP mouse model of parkinsonism^19^.

### Dopaminergic Markers

**Figure 8:**
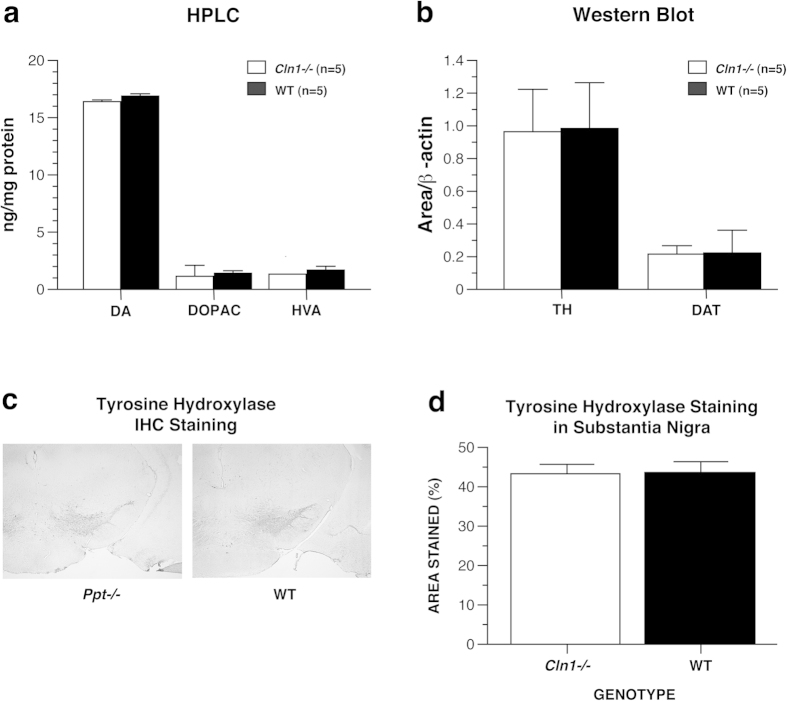
Older *Cln1*^*−/−*^ and WT mice did not differ with regard to biochemical markers of Parkinson’s disease. As measured by high performance liquid chromatography, there were no differences in the levels of dopamine, DOPAC, and HVA between groups (**a**). Levels of tyrosine hydroxylase and DAT, as measured by western blot, were similar between groups (**b**). Panels show coronal sections stained for TH (**c**). Groups did not differ with regard to TH staining density in the substantia nigra. (**d**). Data are expressed as mean + standard error, *Cln1*^*−/−*^ *n* = 3 animals (a minimum of 3 sections/animal, total of 21 sections evaluated), WT *n* = 3 animals, (a minimum of 5 sections/animal, total of 24 sections evaluated).

To determine whether deficits in the dopaminergic system contributed to the motor deficits observed in *Cln1*^*−/−*^ mice, we measured dopamine (DA) levels as well as its primary breakdown products, dihydroxyphenylacetic acid (DOPAC) and homovanillic acid (HVA) in the corpus striatum by high-performance liquid chromatography. *Cln1*^*−/−*^and WT mice had similar levels of DA, DOPAC, and HVA (Fig. 8a). To further investigate the possibility that the DA system plays a part in the motor/sensorimotor impairments in *Cln1*^*−/−*^ mice, we measured levels of tyrosine hydroxylase (TH) and dopamine transporter (DAT) in the corpus striatum via western blot. Similar to the HPLC results, *Cln1*^*−/−*^ and WT mice had similar levels of TH and DAT (Fig. 8b). Immunohistochemical analyses revealed that TH staining density in the substantia nigra was similar between WT and *Cln1*^*−/−*^ mice (Fig. 8c,d). Taken together, these data indicate that an underlying DA deficiency is not responsible for the motor deficits in the *Cln1*^*−/−*^ model.

## Discussion

The goal of the current study was to evaluate a breadth of behaviors in the *Cln1*^*−/−*^ mouse model to further our understanding of INCL and provide useful behavioral benchmarks for studying disease progression and treatment efficacy. Specifically, we assessed behaviors of 1- to 2-month-old (younger) *Cln1*^*−/−*^ mice, which have limited INCL-related neuropathology, and of older *Cln1*^*−/−*^ mice, which have widespread and severe neuropathology^12^,^20^.

As expected, younger *Cln1*^*−/−*^ mice did not exhibit many gross changes in behavior compared to WT mice. However, the younger *Cln1*^*−/−*^ mice showed subtle but significant deficits on the 60° inclined screen and the walking initiation tests. In addition, these mice traveled a shorter distance than WTs on the normal and complex wheel measures and during the actometer test. These results suggest that some of the earliest observable behavioral changes in *Cln1*^*−/−*^ mice appear to be associated with motor/sensorimotor function. It is difficult to determine the cause of these deficits since there is little or no pathological evidence of disease at this stage. It is possible that *Cln1*^*−/−*^ mice do not have the same physical stamina as WT mice, leading to reduced behavioral output over protracted testing. However, this seems unlikely given the age of the younger mice used here. No differences in smooth muscle or bone tissues have been found in 1-month-old *Cln1*^*−/−*^ mice, and at 3 months there is minimal cardiac or pulmonary pathology^2^.

Although younger *Cln1*^*−/−*^ mice showed subtle but significant motor deficits, their cognitive abilities appeared to be intact. This is evidenced by their control-like levels of performance on the MWM and conditioned fear tests. In addition, the performance of *Cln1*^*−/−*^ mice on the normal and complex running wheels improved with increased training/testing even when they exhibited deficits on these tasks. This suggests that the motor learning capabilities of the *Cln1*^*−/−*^ mice may have been intact, although some of their motor/sensorimotor functions may have been compromised. Interestingly, younger *Cln1*^*−/−*^ mice showed significant delays in walking initiation. This may be analogous to a movement dysfunction characteristic of parkinsonism which has been reported in a patient with NCL^17^. However, this finding was not replicated in the older mice suggesting that the walking initiation deficit of the younger *Cln1*^*−/−*^ mice may have been due to altered emotionality such as increased levels of fear or anxiety-like behaviors. This interpretation is not straightforward however, since there was an absence of corroborating findings involving the center area variables from the 1-h locomotor activity test and during the T/S training component of the conditioned fear testing in the younger mice.

Older *Cln1*^*−/−*^ mice exhibited performance deficits on many behavioral tests that required intact motor/sensorimotor function such as the 90° inclined screen test, general ambulatory activity and vertical rearing frequency, normal and complex wheel running, swimming speeds in the MWM, and stride length measured in the actometer. In general, these findings are consistent with and extend the observations in the younger mice that PPT1*-*deficiency results in progressive impairment of motor/sensorimotor function. It is possible that these differences between older *Cln1*^*−/−*^ and WT mice are due to disease-related pathology. Kielar *et al.*^12^ described significant microglial activation at 5 months of age in this animal model. Microglial activation is a reliable marker of neuroinflammation and neurodegeneration, and Kielar *et al.* (2007) specifically noted this activation in somatosensory barrelfield (SB1) and primary motor (M1) cortices, and various thalamic nuclei. Considering the neuronal loss in these loci accompanied by widespread granular osmiophilic deposits (GROD) accumulation, as well as significant pathology in cerebellar white matter, it is plausible that INCL neuropathology directly accounts for decreased motor/sensorimotor capabilities or motivation to explore a novel environment^2^,^12^,^20^. As mentioned previously, systemic pathology in INCL has been described with significant findings in cardiovascular tissue, bone, and various organs in *Cln1*^*−/−*^ mice over 5 months of age^2^. Peripheral disease including neuropathy^21^, in addition to spinal cord abnormalities^18^, may also affect exploratory and motivated behaviors. The lower levels of activity in the older *Cln1*^*−/−*^ mice may also reflect a general malaise caused by the disease. Alternatively, a significant alteration in emotionality could affect activity. For example, increased anxiety-like behavior can result in mice making fewer entries into, spending less time in, and traveling a shorter distance in the center zone during the 1-hr activity test. Lastly, changes in metabolism could account for some differences in overall activity levels. It has been reported that INCL mice at 5 months of age present with decreased adiposity (about 75% of WT levels), decreased body weight, but increased food intake compared to WT mice^22^. Metabolic rate, reported as oxygen consumption measured by indirect calorimetry, was also significantly lower in INCL mice, though still 90% of WT levels. Future studies will include surveys of metabolic rates as well as adiposity measurements in INCL mice in an effort to better understand alterations in overall activity levels.

Although we have provided substantial evidence that older *Cln1*^*−/−*^ mice have robust impairments in motor/sensorimotor functions, our results suggest that some cognitive functions seem to have been spared, at least up until 5–6 months of age. Older *Cln1*^*−/−*^ mice performed similarly to WT controls during the place (spatial learning) and probe (retention) conditions in the MWM and on the contextual fear component of the conditioned fear test. In contrast to the lack of impairment on these tasks, *Cln1*^*−/−*^ mice were impaired during both types of cued trials in the MWM and during the auditory cue component of the conditioned fear test. The impaired performance of the *Cln1*^*−/−*^ mice during the MWM cued trials is interesting in that differences seemed to be greatest in the earliest trials for each cued condition, although the groups performed almost identically by the end of training on each cued task. There are two likely functional disturbances which may account for these deficits considering the subsequent normal spatial learning and memory performance of the *Cln1*^*−/−*^ mice. First, it is known that *Cln1*^*−/−*^ mice develop severe retinal dysfunction later in life^23^ and some decrement in vision was likely to have been present in the 5–6 month old *Cln1*^*−/−*^ mice of the current study. However, any existing visual deficiency was not sufficient to disrupt performance during place and probe water maze trials which rely heavily on the identification of distal visual cues. If *Cln1*^*−/−*^ mice experienced visual impairments at 5–6 months, it may not have been severe enough to prevent the use of visual cues after the mutant mice learned to adapt appropriately during the cued trials.

To further explore the visual capabilities of *Cln1*^*−/−*^ mice, we evaluated the acuity thresholds of another independent cohort of 7-month-old mice by quantifying their optokinetic response during the VOS test. Briefly, the rationale for including this separate, older cohort was to evaluate whether there was a functional correlate to existing ERG findings of diminished rod/cone function at this age using a more direct test that did not involve learning and memory. Our MWM results indicate that, although the cued trials performance of the *Cln1*^*−/−*^ mice was somewhat impaired at the beginning of testing at 5–6 months of age, they retained a sufficient degree of visual function to use distal cues in navigating to specific locations in the pool. Previous research indicated that *Cln1*^*−/−*^ rod/cone function, as measured by electroretinography (ERG), was 70% that of WT mice at 5–6 months of age, which is consistent with the cued trials findings. Results from ERG testing at 7 months of age showed a continuing decrease in rod/cone function in *Cln1*^*−/−*^ mice such that it was only 40% of that for WT mice^23^ at this age. Our present VOS results reveal that 7-month-old *Cln1*^*−/−*^ mice demonstrate significantly decreased visual acuity thresholds relative to WT mice, thus confirming a degree of functional impairment that would be expected from the ERG findings at this age. Given the impaired sensorimotor functions of the *Cln1*^*−/−*^ mice at this age, one might argue that such deficits could affect the execution of their optokinetic response. While this possibility cannot be ruled out completely, we feel that the use of a translucent cylinder to stabilize the *Cln1*^*−/−*^ mice on the test platform allowed the observer to more accurately detect the optokinetic response, and the fact that all mutant and WT mice exhibited the response suggests that the mutant mice still maintained the capacity to respond to the rotating grating.

Another possible functional disturbance affecting cued learning performance in older *Cln1*^*−/−*^ mice might have been slight deficits in procedural learning that dissipated with continued training. Another curious, possibly related, MWM finding in the older mice was the difference in swimming speeds between groups during the cued trials compared to the lack of group differences observed on this variable during the place trials. Specifically, the older *Cln1*^*−/−*^ mice swam slowly compared to the WT group up until the last cued trial and then swam at WT-like speeds during the place and probe trials. The cued protocol used in the present study was twice as long as our typical protocol because we wanted to provide a more rigorous test of visually-guided behavior given our knowledge about the eventual retinal dysfunction in older *Cln1*^*−/−*^ mice. The extended cued training apparently provided sufficient time for the older *Cln1*^*−/−*^ mice to adapt to any visual, motor/sensorimotor, motivational or procedural learning/memory impairments, such that they could demonstrate intact spatial learning and memory capabilities when tested during the subsequent place and probe conditions. This underscores an important procedural issue of developing protocols which may allow mutant mice adequate periods of adaptation to possibly overcome peripherally- and/or centrally-mediated deficits which might confound interpretation of the main behavioral variables of interest.

Other behavioral results that relate to the cognitive capabilities of older *Cln1*^*−/−*^ mice are the data from the conditioned fear test. Importantly, analysis of the contextual fear data (day 2) did not reveal any significant effects involving genotype, indicating that this type of fear conditioning was not impaired in the older *Cln1*^*−/−*^ mice. This is consistent with the finding that spatial learning and memory were intact in these mice and the idea that the hippocampus, which is thought to play a major role in mediating these types of learning and conditioning, may be relatively spared of the pathophysiology associated with PPT deficiency. In contrast to the lack of impairment in contextual fear conditioning observed in the older *Cln1*^*−/−*^ mice were the robust deficits they exhibited during the stages of auditory cue testing. The degree of decreased freezing levels in the older *Cln1*^*−/−*^ mice during exposure to the auditory cue on day 3 is particularly noteworthy considering that these mice exhibited significantly increased levels of freezing during the tone/shock training on day 1 as well as during the 2-min altered context period on day 3 preceding the auditory cue test. Two likely sources for the impaired performance of the older *Cln1*^*−/−*^ mice on this test are compromised auditory cue conditioning and deficits in auditory system processing. Neither has been extensively studied in human INCL patients and therefore it is difficult for us to speculate without supplementary studies. For example, assessing the acoustic startle response and prepulse inhibition of startle along with measuring auditory brainstem evoked potentials would provide extensive information on auditory system function in older *Cln1*^*−/−*^ mice. Also, auditory cue conditioning could be studied further by including additional control groups [e.g., random presentations of the tone (CS) and footshock (US); shock alone, or no shock] to the procedure to distinguish “true” conditioning deficits from other artifactual influences. Additionally, possible amygdalar pathology, an area not currently examined in the INCL literature, may play a role in the impaired auditory cue performance of the *Cln1*^*−/−*^ mice.

We acknowledge that aspects of prior experience including the sequence of the behavioral tests may have had an effect on the outcomes of various measures. However, careful consideration was given to the experimental design of our studies and to developing a test sequence that would allow us to characterize the progression of functional impairments with age in *Cln1*^*−/−*^ mice, but minimize the “carry-over effects” from one test procedure to others. First, we eliminated any effects of prior handling and test experience in the younger mice from affecting behavioral performance in the older mice by using a cross-sectional design involving two independent cohorts of mice instead of using a longitudinal approach. Secondly, we prevented any stress effects from exposure to the footshock used in the conditioned fear procedure from having an impact on any other behaviors by scheduling that test to be the last one conducted for a given cohort. Thirdly, we limited the number of behavioral tests to essential control measures (1-h locomotor activity; sensorimotor battery) before conducting the first cognitive (MWM) test. In summary, the profile of behavioral results across the two ages of the *Cln1*^*−/−*^ mice suggests that our experimental design, including the test sequence, was more than adequate in allowing us to demonstrate a frank progression of motor/sensorimotor impairment with relative preservation of cognitive function in these mutant mice.

Although there are disturbances in several peripheral and central functions that may account for the motor/sensorimotor deficits observed in *Cln1*^*−/−*^ mice as previously described, a subset of these (shortened stride length, walking initiation deficits, and slowed movement) suggested possible Parkinson-like influences. For example, decreased stride length is a characteristic seen consistently in the MPTP mouse model of parkinsonism^19^,^24^. Gait abnormalities, delayed initiation of movement, and generally slowed movements have been described in patients with NCL^14^,^15^,^16^,^17^,^18^ and are typical motor deficits associated with parkinsonism and Parkinson’s disease (PD). Our observations of Parkinson-like deficits in our INCL model provided a rationale for assessing nigrostriatal DA involvement in these movement impairments of the *Cln1*^*−/−*^ mouse by quantifying levels of TH in the substantia nigra as well as levels of DA, DOPAC, HVA, TH, and DAT in the corpus striatum. However, we found no differences between genotypes in expression of TH, DA, DOPAC, HVA, or DAT in the nigrostriatal pathway.

There is a growing body of literature associating LSDs with Parkinson’s disease. Shachar and colleagues (2011) compiled a review of case studies through 2011 that included a Parkinson association with numerous LSDs including Gaucher disease, Niemann-Pick C, juvenile NCL, older NCL, and infantile NCL^25^. Observations included bradykinesia, resting tremor, and rigidity as well as synuclein accumulation in the nigrostriatal pathway and Lewy body inclusions. In addition, a recent case study reported that freezing of gait, an abnormality associated with disturbed initiation of movement and altered stride pattern, is the first motor manifestation in late infantile variant NCL^17^. Though we examined multiple facets of the nigrostriatal DA system for evidence of Parkinson-like pathology to explain the motor/sensorimotor deficits in our *Cln1*^*−/−*^ mice, our data are not consistent with the notion that dopaminergic deficiency underlies the motor disturbances measured in this model. Instead, it seems likely that such motor abnormalities are associated with progressive disease pathology in M1, SB1, cerebellum, and thalamus. It is imperative that caution be exercised when claiming an association of PD with LSDs, as this may affect understanding of the disease as well as treatment and management decisions.

The current study contributes several valuable findings to the INCL literature. First, we have identified several useful tools for monitoring the progression of INCL and for evaluating a response to therapy. We have also demonstrated that while INCL mice show significant motor/sensorimotor deficits and CNS pathology, many of their cognitive capabilities remain intact, at least for the ages evaluated in the present study. Finally, the DA/TH data presented here highlight the need for biochemical and histological confirmation before an association between PD and INCL can be established.

## Materials and Methods

### Animals

The *Cln1*^*−/−*^ mouse was created through a targeted disruption strategy that eliminates the last exon of the murine *Cln1* gene^10^. This targeted mutation was backcrossed to C57BL/6 mice for > 10 generations, and from the final generation colonies of *Cln1*^*−/−*^ and *Cln1*^+/+^homozygotes were split off and maintained by MSS. Male and female mice of each genotype (N = 30 PPT1-deficient; *Cln1*^*−/−*^, N = 22 wild-type;WT) served as subjects in the experiment. WT and *Cln1*^*−/−*^ mice were not littermates. All animals were housed in an animal facility at the Washington University School of Medicine (St. Louis, MO) under a 12 hr light/dark cycle and were provided food and water *ad libitum.* Behavioral tests were conducted during the light cycle.

### Ethics Statement

All animal procedures were approved by the Institutional Animal Studies Commi-ttee at Washington University School of Medicine and were in accordance with the guidelines of the National Institutes of Health.

### Experimental Design

A cross-sectional design was used which involved two cohorts of *Cln1*^*−/−*^ and WT mice. In one cohort, behavioral testing was initiated at a mean age of post-natal day (PND) 27, and testing continued through PND 65. Although testing occurred up until early adulthood, this cohort will be referred to as the “younger” cohort. In the younger cohort, the sample size for the *Cln1*^*−/−*^ group was n = 16 (4 females; 12 males), while for the WT mice n = 11, (6 females, 5 males). In an “older” cohort of mice, behavioral testing began at PND 147 and continued through to PND 185. In this older cohort, the sample sizes were n = 14 for the *Cln1*^*−/−*^ group (5 females; 9 males) and n = 11 for the WT control mice (7 females; 4 males). The mice were euthanized at the end of testing for each cohort.

A separate, older cohort of mice (7 months of age, N = 18; 9 WT and 9 *Cln1*^*−/−*^) was tested for visual acuity using the VOS procedure to evaluate whether there was a functional correlate to existing ERG findings showing greatly diminished rod/cone function at this age. The VOS test provided a more direct assessment of visual system function that did not involve learning and memory.

### Behavioral Testing

#### 1-hr locomotor activity

To examine general activity levels and possible differences in emotionality, mice were evaluated over a 1-hr period in transparent polystyrene enclosures measuring 47.6 × 25.4 × 20.6 cm high. Our procedure has been described previously^26^. Each enclosure was surrounded by frames containing pairs of photobeams which were monitored by computer software (MotorMonitor, Kinder Scientific LLC, Poway, CA). General activity variables included total ambulations (whole body movements), vertical rearing frequency, distance traveled in a 5.5 cm wide peripheral zone, and time spent resting. Emotionality measures included time spent in, distance travelled in, and number of entries into a 33 × 11 cm central zone.

#### Sensorimotor battery

*Cln1*^*−/−*^ mice were assessed on a battery of sensorimotor tests to evaluate balance, strength, and/or coordination. The test included walking initiation, ledge, platform, pole, 60° and 90° inclined screens, and inverted screen. All tests have been described previously^26^ and mice were tested twice on each apparatus, and a mean of the two scores was used for analysis.

#### Morris water maze (MWM)

The MWM test was used to evaluate spatial learning and memory in the mice utilizing a slightly modified version of our previously published procedure^27^. The protocol included cued, place, and probe trials. Testing took place in a round pool (118 cm diameter) containing water made opaque with non-toxic white tempura paint. All trials were monitored through a live video feed by computer software (Any-maze, Stoelting Co., Wood Dale, IL) which calculated swim speed, escape path length, escape latency, and time and distance spent in each of the four quadrants of the pool. The maximum score for all water maze trials was 60 s.

Cued trials were conducted first to determine whether nonassociative deficits (e.g., visual or sensorimotor disturbances, or alterations in motivation) were likely to affect swimming performance and confound interpretation of the acquisition data collected during the place trials. Mice received 4 cued trials per day for a total of 4 consecutive days. On days 1 and 2, mice were placed in the quadrant directly opposite a submerged platform marked with a visible cue (red tennis ball on top of a pole). The platform was moved to a different location for each trial (60 s intertrial interval) within a day and there were very few distal cues available during this time, both of which limited spatial learning. The same protocol was used during days 3 and 4 of the cued trials except that the tennis ball was removed from the pole, leaving only the latter to serve as the cue. This was done to decrease the salience of the cue and provide a more difficult test for visually-guided behavior. Escape path length and latency and swimming speeds were used to evaluate performance during the cued and place conditions.

Place trials were performed the day after completing the cued trials. In the presence of salient, stationary distal cues to facilitate acquisition (spatial learning), mice were evaluated on their ability to learn the location of a hidden, unmarked platform. Four place trials per day were administered for 5 consecutive days during which the platform remained in the same location for all trials. The mice were released from 4 different locations each day. The daily protocol involved administering 2 sets of 2 trials each, with sets being separated by approximately 1 hr.

A single probe trial was administered approximately 1 hr after completion of place trials on the fifth day. During the probe trial, the platform was removed from the pool and the animals placed in the quadrant directly opposite the former platform location. Mice were allowed to explore the water maze for 60 s during which time various aspects of their search behaviors for the platform were quantified. The number of times a mouse passed directly over the platform location (platform crossings), the time spent in the target quadrant that had contained the platform, and spatial bias were used to evaluate retention performance during the probe trial. Spatial bias refers to a mouse spending significantly more time in the target quadrant compared to the times it spent in each of the other quadrants.

#### Visual acuity

An independent cohort of 7-month old *Cln1*^*−/−*^ and WT mice was evaluated for visual acuity using the VOS test as described previously^28^,^29^. Briefly, the apparatus consisted of a virtual cylinder-like display comprised of a vertical sine wave grating projected in three-dimensional (3-D) space on computer monitors. The 4 monitors were arranged in a quadrangle around a central circular platform, forming a square arena (46 cm × 46 cm). A camera (FireWire iSight; Apple Computer Corp., Mountain View, CA, USA) was positioned directly above the platform to allow observation of mouse behavior. After a mouse was placed on the central platform, light and dark bars were projected on the monitors to give the appearance of bars rotating around the mouse. The virtual rotational motion of these bars induced optokinetic head/body tracking movements. Thresholds for visuospatial acuity were generated by increasing the frequency of the sine waves until the optokinetic response was no longer observed, indicating that the animal no longer distinguished the individual bars rotating around it. The speed of rotation and geometry of the projected bars were controlled by the system software. The dependent variable was the highest grating (in cycles per degree; cyc/deg) at which the animal could discriminate between light and dark rotating lines before failing to display optokinetic responses. The sensorimotor impairments of the *Cln1*^*−/−*^ mice at this age made it difficult for them to remain on the test platform. For this reason, a translucent *Plexiglas* cylinder (6.5 cm diam; 21 cm high) was placed over the circular platform when testing both the *Cln1*^*−/−*^ and WT mice. This modification allowed the mutant mice to remain on the platform during the entire test trial but did not restrict their optokinetic response and also facilitated the observer’s judgment concerning the presence of the response.

#### Normal and complex running wheel

Mice were evaluated for voluntary wheel running activity as well as on their performance of a difficult sensorimotor (complex wheel) task which required fine motor coordination between fore- and hind-limbs^30^.

Mice were first tested on a normal running wheel (Mouse Motor Skill Sequences Activity Wheel, Lafayette Instrument, Lafayette, IN) for 1 hr on 5 consecutive days (1 trial/day) to establish baseline voluntary wheel running activity. The normal activity wheel contained 38 consecutive rungs (0.4 cm in diameter) that were spaced 0.614 cm apart. Mice were allowed to explore the wheel and activity chamber for 1 hr on 5 consecutive days. Dependent variables included average speed, maximum speed, time spent running on the wheel, distance, and time spent not running on the wheel.

A complex running wheel was created by removing some of the wheel rungs thus creating unequal spaces between the rungs of the wheel (0.614, 1.6, or 2.6 cm^30^). The mice received 5 consecutive days of acquisition training on the complex wheel followed by two days of no wheel running and then 5 consecutive days of performance testing on the complex wheel, using the same dependent variables that were utilized during the normal activity wheel phase.

#### Conditioned fear

Fear conditioning capabilities were evaluated in the mice over a 3-day test period using a previously described protocol^27^. This test was administered last in the sequence of behavioral measures in an effort to avoid any “carry-over effects” on other behaviors of interest as a result of the mice having been exposed to footshock during the procedure. Testing took place in two *Plexiglas* conditioning chambers, each differing in terms of visual, tactile, and olfactory cues. On the first day *(tone-shock training*), each mouse was placed in one *Plexiglas* chamber for a 5-min trial during which freezing behavior was quantified by computer software (FreezeFrame, Coulborn Instruments, Whitehall, PA). The first 2 min served as a baseline period, after which a conditioned stimulus (CS) in the form of an 80 dB tone (white noise) was presented for 20 s. During the last second of the CS, a 1.0 mA foot shock (unconditioned stimulus; US) was administered. This pairing was repeated for each of the next 2 min for a total of 3 CS-US pairings. Twenty-four hr later, each mouse was placed back into the same chamber used on the first day of testing, for an 8-min trial during which no CS or US was presented. Again, freezing behavior was quantified in order to assess the amount of *contextual fear* conditioning that occurred in each group. Twenty-four hr after this trial, mice were evaluated on the auditory cue test. Each mouse was placed in the other *Plexiglas* chamber, which contained different cues, and freezing behavior was quantified for 10 min in this “altered context.” The first 2 min served as a baseline period, followed by 8 min when the *auditory cue* (CS) was continuously presented. The dependent variable for each trial was the percent of time that the mouse spent freezing.

#### Actometer

To assess exploratory behavior, gait and other movement-related functions, each mouse was tested in a force plate actometer^31^,^32^. Briefly, the apparatus is a square load plate (42 × 42 cm) that sits atop 4 load cell transducers (Honeywell/Sensotec, Columbus, OH) and is surrounded by 4 *Plexiglas* walls measuring 43 × 30.5 cm. A mouse is placed in the center of the chamber and allowed to explore for 20 min. Dependent variables included distance travelled, low mobility bouts (number of instances the animal remains in the same location for 10 s at a time), and parameters related to gait analysis. For gait analysis, each 20-min recording session was visualized with a scrolling graphics program that enabled the user to identify the distinctive rhythmic force time-wave forms (acquired at 100 samples/s) that accompany locomotion or “runs”^32^. Also displayed were the spatial coordinates, x and y, of the lateral movements as a function of time. With a mouse-controlled cursor the program user marked the beginning and ending of 20 separate runs that were 2.5 or more strides long. For each mouse, 10 runs were taken from the beginning of the recording session and 10 additional runs were taken from the end of the session and counting back toward the middle. The force-time information and corresponding spatial information were then subjected to a series of calculations that produced stride length (mm) and stride rate (Hz, i.e., strides/s). For a run to qualify for inclusion in the analysis it had to have a nearly straight-line trajectory between its starting and ending points, no pausing, and be comprised of 2.5 or more strides.

### Dopaminergic markers

#### Tissue collection

Mice were deeply anesthetized and transcardially perfused with ice-cold phosphate buffered saline. A coronal section through the striatum was cut using an ice-cold Zivic rodent brain matrix. The striatum from each hemisphere was isolated and weighed separately. For catecholamine analysis, one unilateral striatum was homogenized in 50-fold (weight: volume) ice-cold PCA buffer (0.1 N perchloric acid, 0.2 mM sodium metabisulfite). The homogenized tissue was briefly sonicated with a Branson Sonifier Cell Disruptor (Danbury, CT) and centrifuged at 14,000 g for 10 min at 4 °C. The supernatant was stored at −80 °C. For protein analysis, the remaining unilateral striatum was homogenized in Ripa buffer (50 mM Tris, pH 8.0; 150 mM sodium chloride; 1.0% Triton X-100; 0.5% sodium deoxycholate; 0.1% sodium dodecyl sulfate) containing Roche ‘Complete’ Protease inhibitor cocktail. The homogenate was centrifuged at 10,000 g for 5 min at 4 °C. The supernatant was stored at −80 °C.

#### Dopamine, DOPAC, and HVA analysis

High pressure liquid chromatography was performed using an ESA Coulochem III electrochemical detector (Bedford, MA) on a MD-150 × 3.2 mm column with MD-TM hplc buffer (75 mM sodium dihydrogen phosphate, monohydrate; 1.7 mM 1-octanesulfonic acid, sodium salt; 100 μL/L triethylamine; 25 μM EDTA-tetrasodium salt, tetrahydrate; 10% acetonitrile; pH 3.0).

The samples were diluted with MD-TM hplc buffer and filtered through a 0.22 μM syringe filter to remove any fine particulates. 100 μL was injected onto the HPLC and eluted with MD-TM mobile phase at a rate of 0.6 mL/min. Concentrations of dopamine, DOPAC, and HVA in the striatal samples were obtained by comparing to a series of catecholamine standards. The levels of dopamine and its metabolites were normalized to tissue weights.

#### Tyrosine hydroxylase and dopamine transporter measurements

Striatal tissue was homogenized in Ripa buffer (50 mM Tris, pH 8.0; 150 mM sodium chloride; 1.0% Triton X-100; 0.5% sodium deoxycholate; 0.1% sodium dodecyl sulfate) containing Roche ‘Complete’ Protease inhibitor cocktail. Protein concentration was determined using the Coomassie blue dye-binding assay (BioRad). Striatal lysates were separated using SDS-PAGE and then transferred to polyvinylidene difluoride membranes (BioRad, Hercules, CA). Blots were probed with the following primary antibodies: mouse anti-tyrosine hydroxylase (TH, MAB318,EMD Millipore), rat anti-dopamine transporter (DAT, MAB369, EMD Millipore), and mouse anti-β-actin (A5441, Sigma-Aldrich). The following secondary antibodies were used: Cy5-conjugated donkey anti-mouse IgG (Jackson Immunoresearch), peroxidase conjugated goat anti-mouse IgG (Sigma-Aldrich), and peroxidase conjugated goat anti-rat IgG (Sigma-Aldrich).

Immunohistochemical staining for TH was performed as previously described^33^. Briefly, 16-micrometer floating coronal sections through the substantia nigra were stained for TH (1:500, Abcam). Sections were mounted onto slides, blinded, and imaged by light microscopy. Forty-five sections from the rostral to caudal aspects of the substantia nigra (1.06 to 4.16 nm caudal of Bregma) were stained and photographed^34^. All images were captured using identical settings. The area fraction of TH staining was analyzed by Image J software^35^.

### Statistical Analyses

All statistical analyses were conducted using PASW Statistics 18, Release Version 18.0.0 (SPSS, Inc., 2009, Chicago, IL). Means and standard errors were computed for each variable. Analyses included, where appropriate, factorial ANOVAs, including repeated measures ANOVAs (rmANOVAs), and one-way ANOVAs. All ANOVA models contained genotype as a between-subjects variable. The rmANOVA models also typically contained either one within-subjects variable (e.g., blocks of trials) or two within-subjects variables (e.g., trials and sessions). Simple main effects were calculated in the case of a significant interaction. In the event of a violation of sphericity as measured by Mauchly’s sphericity test, the F statistic, degrees of freedom, and *p*-value were all corrected via the Greenhouse-Geisser or Huynh-Feldt method in accordance with accepted guidelines^36^. For a significant F value for main and simple effects, pairwise comparisons were used to compare means over the repeated measure. Probability values of *p* < 0.05 denoted significance for all analyses, and multiple comparisons were Bonferroni adjusted.

## Additional Information

**How to cite this article**: Dearborn, J. T. *et al.* Comprehensive functional characterization of murine infantile Batten disease including Parkinson-like behavior and dopaminergic markers. *Sci. Rep.* **5**, 12752; doi: 10.1038/srep12752 (2015).
